# The risk and resilience factors associated with secondary traumatic stress in school personnel: A scoping review

**DOI:** 10.1111/bjep.70101

**Published:** 2026-07-02

**Authors:** Paul Gordon, Lisa Jane Rodgers, Martin Dempster, Donncha Hanna

**Affiliations:** ^1^ School of Psychology Queen's University Belfast Belfast UK

**Keywords:** compassion fatigue, resilience, risk factors, school personnel, secondary traumatic stress, teacher well‐being

## Abstract

**Background:**

School personnel bear the emotional burden of students' trauma. Secondary traumatic stress (STS), resulting from indirect trauma exposure, impacts well‐being and job performance through PTSD‐like symptoms, making addressing STS in schools a critical priority.

**Aims:**

This scoping review mapped and synthesized risk and resilience factors associated with STS in school staff and tabulated the instruments used to assess STS in this population.

**Sample:**

A search across five databases yielded 1238 records; 48 studies met the inclusion criteria following dual independent reviews.

**Methods:**

This review was guided by the Joanna Briggs Institute (JBI) methodology and followed PRISMA‐ScR guidelines for reporting.

**Results:**

Key resilience factors included cognitive and emotional resources (e.g., metacognition) and leadership support within trauma‐informed organizational climates. Conversely, student trauma exposure, personal trauma history, and specific professional demographics consistently increased STS risk. The Professional Quality of Life (ProQOL) scale and Secondary Traumatic Stress Scale (STSS) were the most utilized measures.

**Conclusions:**

STS is an organizational issue as much as an individual one. Fostering personal cognitive‐emotional resources and cultivating trauma‐informed leadership are critical levers for mitigating STS. However, a considerable reliance on social work‐ and healthcare‐derived STS measures was evident. These findings identify potential targets for interventions and policies aimed at improving well‐being and can inform future meta‐analyses.

## INTRODUCTION

Approximately, 50% of individuals experience at least one traumatic event or adverse childhood experience (ACE) by the age of 18 (Bellis et al., 2014; Lewis et al., 2019). The effects of these experiences have been linked to short‐ and long‐term consequences for children, such as poor academic performance and behaviour, negative mental and physical health, and other adverse outcomes that may extend into adulthood (Christian‐Brandt et al., 2020). Given the widespread exposure to trauma among school‐aged children, school personnel, such as teachers and school mental health support staff, are tasked with addressing childhood trauma on the ‘front lines’ of classrooms and schools (Christian‐Brandt et al., 2020). Trauma‐affected youth often demonstrate behavioural issues, which frequently lead to teacher–student conflict and re‐traumatization, contributing to teachers' occupational stress, burnout, and motivation to leave the teaching field (Fazel et al., 2014). Furthermore, the extent of the exposure to students' negative emotional and mental health states is such that it has prompted some authors to characterize educators as ‘first responders’ to students in their classroom (Conroy et al., 2024; Gunawardena et al., 2024). This type of exposure is potentially significant to staff retention, as there was a 44% increase in teachers considering leaving the field between the 2021/22 and 2022/23 school years (McLean et al., 2024), with poor well‐being, including stress‐related issues and student behaviour, consistently cited as reasons for departure (Doan et al., 2023, 2024).

Educators spend a significant amount of time with students each week and are uniquely positioned to hear or observe narratives of violence and accounts of traumatic events, such as through students' graphic writings or drawings (Hydon et al., 2015). Additionally, some school staff act as a link between families and mental health services, often leading them to bear the emotional burden of their students' experiences (Alisic et al., 2012). This professional closeness to suffering can cause staff to absorb the distress and trauma of their students, leading to symptoms such as intrusive thoughts, irritability, and emotional numbing that can mirror those of post‐traumatic stress disorder (PTSD) (Alisic, 2012; Caringi et al., 2015; Christian‐Brandt et al., 2020; Hydon et al., 2015). This phenomenon is known as *Secondary Traumatic Stress* (STS; Figley, 1995).

### STS theories, definitions, and measures

To date, though investigation into secondary trauma has been growing, there has not been a single definition for STS adopted with unanimity (Sprang et al., 2019). Instead, several terms and definitions have been used interchangeably to describe the same construct, based on three dominant theories or models.

First, Figley (1995) defines STS as the natural emotions and behaviours resulting from knowing of a significant other's traumatic event, including the stress from helping or wanting to help the person. Similarly, secondary traumatic stress disorder (STSD) is the syndrome derived from STS, with symptoms ‘nearly identical to PTSD’ (Figley, 1995, p. 8). In other words, this theory poses that STS is the natural result of helping or wanting to help a significant other, presenting on a continuum from mild to clinically significant, potentially reaching symptoms that mirror PTSD, which requires symptoms of re‐experiencing the event, avoiding trauma‐related stimuli, starting or worsening negative thoughts or feelings about the self or others, and changes in arousal for clinical diagnosis (American Psychiatric Association, 2013). Proponents of Figley's (1995) theory consider STS and compassion fatigue to be interchangeable, as they both potentially produce the same responses as PTSD (Ludick & Figley, 2017). Recent amendments to the Diagnostic and Statistical Manual of Mental Disorders, 5th Edition (DSM‐5; American Psychiatric Association, 2013) affirm that secondary exposure to trauma, in some cases, may meet PTSD diagnosis criteria. It is this potential overlap between STS and PTSD that warrants the current investigation of specific factors related to risk and resilience to STS in school staff.

Second, Stamm (2010) conceptualized STS as part of the split model of Professional Quality of Life (ProQOL). This model revised Figley's (1995) work on compassion fatigue by distinguishing two sub‐constructs of this negative consequence: burnout and STS. Compassion Satisfaction is the other branch of the ProQOL model, which constitutes the positive effects of helping traumatized individuals (Stamm, 2010). Moreover, this theoretical viewpoint defines STS as a negative affective state driven by fear and work‐related trauma, arising from exposure to the traumatic experiences of others, differing from the burnout characteristic of work‐related exhaustion, not necessarily pre‐empted by trauma exposure (Stamm, 2010). Given the conceptual distinction between burnout and STS, this review focuses primarily on STS, which requires secondary exposure to trauma, whereas burnout does not.

Third, McCann and Pearlman (1990) viewed the effects of secondary exposure to trauma through the constructivist self‐development theory (CSDT) and conceptualized it as vicarious traumatization (VT). Specifically, VT is said to impact the professional over time by changing their belief system and cognitive schemas about themselves and others (McCann & Pearlman, 1990). It is important to note that VT has been found to share risk factors with STS (Baird & Kracen, 2006; Gusler et al., 2025; Hensel et al., 2015) and has been used interchangeably across the literature, though being separate from STS (Bride et al., 2004; Ludick & Figley, 2017; Sprang et al., 2019). A recent network analysis supported this distinction, finding that ‘STS and VT are distinct but related phenomena’, yet a strong connection between the two remains, especially with intrusion symptoms (Gusler et al., 2025, p. 7). Consequently, VT, compassion fatigue, and STS were included in this scoping review's search strategy as related outcomes of secondary trauma to ensure that the complex relationship among these constructs was captured.

Given the lack of consensus on terms and definitions, the current review utilized a combination of Figley's (1995) and Stamm's (2010) conceptualization as the operational definition of STS. For this review, studies that conceptualized or defined STS as equivalent to compassion fatigue (Figley, 1995) or as a component or sub‐construct of compassion fatigue (Stamm, 2010) met the operational definition of STS for inclusion. Furthermore, as preregistered in this review's protocol, compassion satisfaction was included as a covariate only if STS, compassion fatigue, or VT was investigated as a related outcome, and all other inclusion criteria were met.

### Measuring STS

Several measures have been used across the literature to capture attributes of secondary trauma, including the compassion fatigue self‐test (Figley, 1995) and the Trauma Attachment Belief Scale (Pearlman, 2003). However, the two predominant measures used to assess STS in the current literature and context are summarized in this section. The Professional Quality of Life Scale (ProQOL; Stamm, 2010) was developed and revised from a version of Figley's (1995) compassion fatigue self‐test. This measure corresponds with the split model of compassion satisfaction – compassion fatigue discussed earlier, where STS and burnout are the two sub‐components of compassion fatigue, the adverse effects of helping traumatized individuals, and compassion satisfaction represents the positive effects. Second, the Secondary Traumatic Stress Scale (STSS; Bride et al., 2004) was developed in line with DSM‐5 symptoms and diagnostic criteria for PTSD, though tailored for indirect trauma.

STSS and ProQOL measures have been critiqued regarding their validity and utility in some contexts (Sprang et al., 2019). Studies using ProQOL have removed items after factor analysis showed structure issues (Craig & Sprang, 2010; Shen et al., 2015), and others have suggested poor reliability and validity of its STS sub‐scale (Fleckman et al., 2022; Gusler et al., 2025; Hemsworth et al., 2018; Heritage et al., 2018). It is also suggested that since STS is an ecological construct based on systems theory, the STSS may be too narrowly focused as it only assesses traumatic stress symptoms, thereby missing other domains (Sprang et al., 2019). Moreover, these measures are not consistently interpreted (Lepore, 2016; Ormiston et al., 2022) and are sometimes used to measure constructs that appear inconsistent with their design. For example, an influential dissertation by Abraham‐Cook (2012) describes, ‘in the current study, compassion fatigue was measured using the Secondary Traumatic Stress scale of the ProQOL’ (Abraham‐Cook, 2012, p. 12). Furthermore, a peer‐reviewed study using the STSS characterized its purpose as assessing ‘the three primary symptoms of compassion fatigue’ (Woloshyn et al., 2023, p. 496), illustrating the variability in current interpretations of the measured construct. Therefore, outlining the measures used to assess STS in school personnel via this review is an important step towards understanding the phenomenon in this context.

### Secondary traumatic stress (STS) in school personnel

While STS has primarily been studied in other helping professions, such as nurses and less so in school personnel (Christian‐Brandt et al., 2020), it has been widely suggested across literature that STS interrupts teachers' capacities for recognizing the emotional needs of their students (Simon et al., 2022) and can impact educators' acceptance of trauma‐informed practices in their schools (Christian‐Brandt et al., 2020). Trauma‐informed frameworks and practices (TIP) are system‐level operational models that acknowledge the prevalence, varied symptoms, and long‐lasting behavioural and health effects of trauma (Champine et al., 2022), and implement practices that respond to trauma and minimize re‐traumatization (Brown, Carlisle, et al., 2022; Brown, Freedle, et al., 2022; Substance Abuse and Mental Health Services Administration [SAMHSA], 2014). Since the evidence above indicates that STS affects school personnel's acceptance of TIP, it is critical to know the factors that predict STS so educational organizations can potentially maximize the adoption of TIP. Moreover, Simon et al. (2022) found that teachers who scored higher on self‐reported STS symptoms also rated students' socio‐emotional problems in class higher than teachers with low STS symptoms, which highlights the correlation between STS and how teachers may perceive students' behaviour as more problematic.

Attaining broad prevalence rates of STS in school staff has been limited by inconsistencies in conceptualization and measurement (Sprang et al., 2019). However, a recent systematic review reported snapshots of STS ‘at risk’ prevalence rates among teacher samples, with studies reporting ‘moderate to high rates’ and ‘the low end of the moderate range’ (Ormiston et al., 2022, p. 811). It is important to investigate constructs like STS, particularly the risk and protective factors in this population, as it has been shown above that STS has been reported in school staff samples, the potential for engaging with traumatized students is at least 50% in some classrooms, and the effects could lead to poor job efficacy and well‐being for staff. Furthermore, research has linked teachers' well‐being with student engagement, behaviour, and academic success (Havik & Westergård, 2020; Jennings & Greenberg, 2009; Moreira et al., 2020; Roorda et al., 2017; Schonert‐Reichl, 2017; Simon et al., 2022). To fully realize schools' protective potential, education systems must address the well‐being of school personnel, which includes the potential risk of STS. Understanding which factors predict STS and those that could potentially mitigate it in this population is a necessary path towards promoting health and well‐being among school staff.

### Study aim

Underpinned by two complementary theoretical viewpoints, this scoping review investigates the construct of STS within the bifurcated model of compassion fatigue and compassion satisfaction (Stamm, 2010). Specifically, the primary aim is to map and synthesize the explanatory factors of STS in this population and categorize them into groups based on the Refined Trauma Model for Trauma Workers, as described by MacRitchie and Leibowitz (2010). A second aim of this review is to tabulate the instruments used in quantitative studies to assess STS in this population. The review seeks to delineate evidence of robust explanatory factors and to highlight existing gaps, or instances where evidence is currently insufficient or conflicting.

## METHOD

We utilized methods guided by the Joanna Briggs Institute (JBI) methodology for evidence synthesis (Peters et al., 2020; Pollock et al., 2023). Reporting was guided by the Preferred Reporting Items for Systematic Reviews and Meta‐Analyses extension for scoping reviews (PRISMA‐ScR; Tricco et al., 2018). This method was selected due to the anticipated mixed state of the studies on this specific population, and our aim of identifying patterns, mapping heterogeneous factors, and disseminating what is known and what is not about this topic. The methodology, including eligibility criteria, search strategy, and data analysis plan, was specified in advance. The protocol for this review was preregistered with the National Institute for Health Research's (NIHR) International Prospective Register of Systematic Reviews (PROSPERO) on May 3, 2024 (ID:CRD42024542965).

### Search strategy

After consulting with institutional research librarians for the most appropriate databases and search terms for the topic, we utilized the following five databases for this review: *PsycINFO*, *ERIC*, *Medline*, and *Web of Science* for peer‐reviewed journals; and *ProQuest Dissertations and Theses Global* for PhD theses/dissertations. The following nested Boolean string served as the primary search: (secondary traumatic stress or STS or compassion fatigue or vicarious trauma* or secondary trauma*) AND (personnel or teacher* or counsel* or social worker* or psychologist* or staff) AND (education* or school*). This nesting technique was critical for filtering out several thousand irrelevant records associated with other ‘STS’ acronyms, such as the academic field of ‘Science, Technology and Society’. Minor adjustments to the nested search string were made based on the limiter logic and database context. For example, because ERIC is an education science resource and automatically stems terms (typically requiring an asterisk on other platforms), we removed the education limiters (education* or school*) in this database to reduce unnecessary limiting. We used broader terms such as ‘staff’ and ‘personnel’ because they reflected the state of the literature and were inclusive enough to encompass roles such as principals. A validation check across all selected databases via a centralized university library platform added only two additional references prior to screening, indicating that the primary search strategy was robust. The final database search occurred in May 2024.

### Eligibility criteria

The population for this study was ‘school personnel,’ specifically teachers, principals, and school mental health support staff who work with students at the secondary level or below. Included studies had to be quantitative and primary research, published in peer‐reviewed journals or as PhD dissertations. Studies must have investigated risk or resilience factors associated with STS in school personnel. Studies investigating compassion fatigue or VT were included if they met the operational definition outlined previously or reported STS as a sub‐scale related to the risk factors. Studies also had to report on quantitative relationships between risk or protective factors and their respective outcome. Additionally, studies must have described and utilized validated measures for evaluating STS or other characterizations of secondary trauma outcomes reported (e.g., compassion fatigue). Statements indicating that either construct, content, criterion, or face validity had been tested and supported sufficed as evidence of a valid measure. No limitations were placed on publication year, geography, or language. Summaries, reviews, studies evaluating interventions, and books were excluded.

### Study selection

Although scoping reviews are sometimes viewed as less restrictive than other reviews, JBI guidelines suggest that reviewers may impose restrictions based on the types of sources most relevant to the aims (Peters et al., 2020). Therefore, we decided a priori to limit sources to quantitative, peer‐reviewed, and doctoral dissertations investigating explanatory factors in the school context, as preregistered. The first author conducted searches of all databases and identified records. The software EndNote (v.21) was used as the primary reference management platform to import, deduplicate, and screen titles and abstracts. After the initial title and abstract screening, two reviewers independently conducted the full‐text assessment of records for inclusion to reduce selection bias. To improve quality, peer‐reviewed studies based on PhD theses/dissertations that used the same dataset were selected over their non‐peer‐reviewed versions. Non‐peer‐reviewed versions were excluded during screening and are indicated in the PRISMA diagram (Figure 1) as ‘duplicated data in report’. We used the web‐based platform Covidence (Veritas Health Innovation, Melbourne, Australia) to track and manage the full‐text review, including resolving conflicts. Inclusion conflicts were resolved by a third member of the research team. Inter‐rater reliability for the full‐text screening was calculated using Cohen's kappa. There was strong agreement between the two raters on study inclusion decisions, κ = .91, which is interpreted as ‘almost perfect’ agreement according to the benchmarks proposed by McHugh (2012).

**FIGURE 1:**
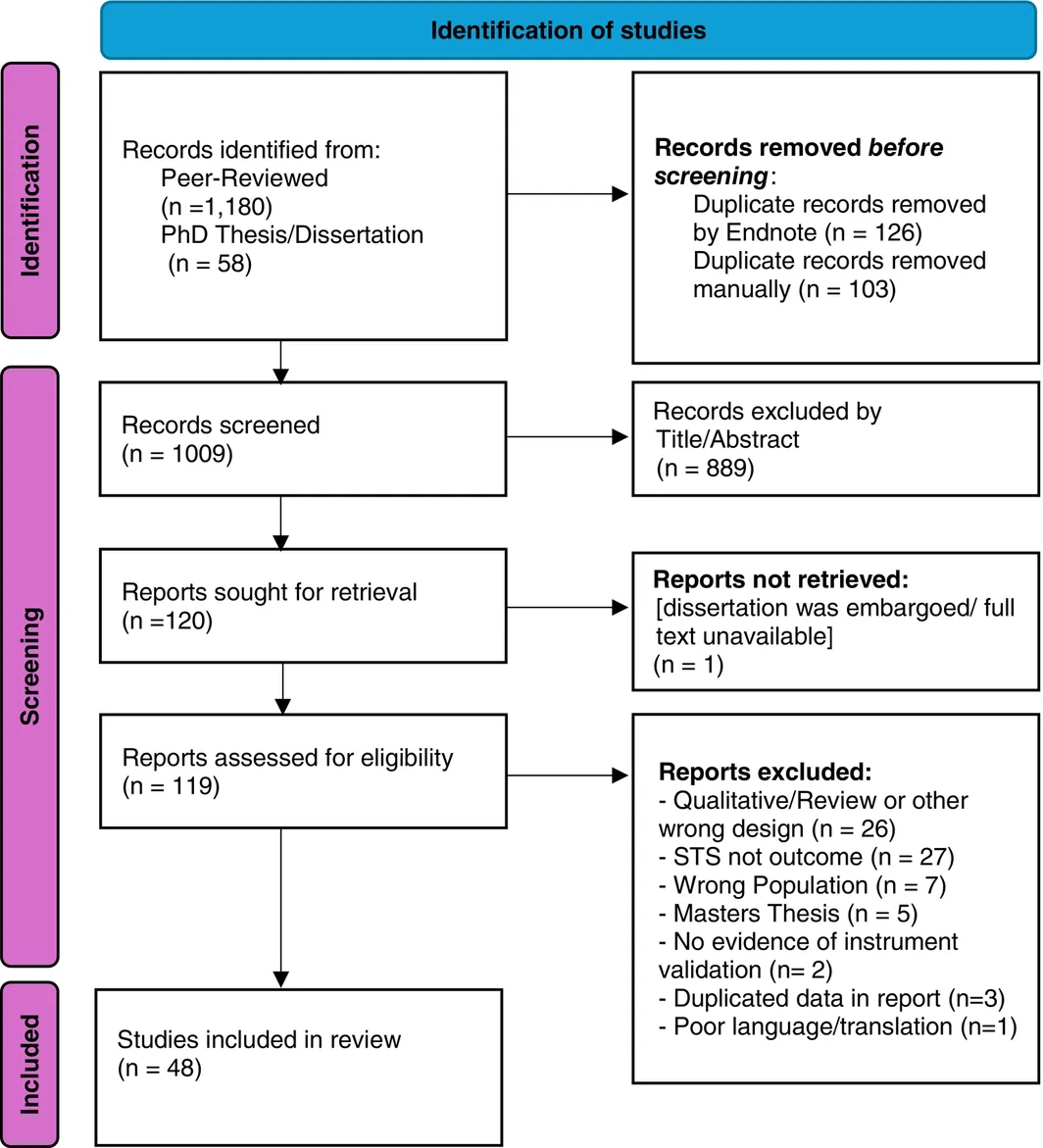
PRISMA flow diagram.

### Data extraction

Studies retained after full‐text eligibility screening were moved to the data extraction area of the Covidence platform, where the first author manually processed them to collate relevant data, as preregistered in the study protocol. This included each study's author, country, population, sample size, instrument, and results related to STS explanatory variables investigated. The extracted data are summarized in Table 1. Data were exported to an Excel (Version 2505) spreadsheet and reviewed by a third member of the research team before moving to the Critical Appraisal of Sources.

**TABLE 1:** Included studies summary.

No.	Study ID	Country	Dissertation/peer‐reviewed	Independent variables	STS instrument used	Secondary trauma measured/reported as	Population	N	Method(s) of analysis	Key results	QuADS score
1	Abraham‐Cook (2012)	US	Dissertation/Thesis	Years exp., trauma hx., STS exp., time mgmt., workload, discipline and motivation, prof. distress, prof. investment, social support, CS, CF	ProQOL	Combined or reported as compassion fatigue (CF)	Pre‐K‐12 educators	111	Pearson's correlation, descriptive, regression	SIG (STS exposure: *r* = .20, β = .17) SIG (Social support: *r* = −.33) SIG (Time mgmt: *r* = .60, β = .22); Discipline and motivation: *r* = .51; Professional distress: *r* = .37; Professional investment: *r* = .41; Work‐related stressors: *r* = .65, β = .39	35
2	Alexander (2024)	US	Dissertation/Thesis	Caseload, % trauma exp. clients, time in direct service	ProQOL	STS distinct variable/outcome	School social workers	53	ANOVA, regression, descriptive	SIG when 50% of caseload (Caseload trauma: *F*_(2,50)_ = 4.37, *b* = 5.90, t (50) = 2.88); NS (Caseload size); NS (Trauma contact)	35
3	Anama‐Green (2022)	US	Peer‐reviewed Journal	Intrapersonal and interpersonal mindfulness	ProQOL	STS distinct variable/outcome	Educators	144	ANOVA, descriptive, odds ratio	SIG (Intrapersonal mindfulness: *r* = −.523)	21
4	Asquith (2023)	US	Dissertation/Thesis	Demographics, years service, plan to leave, teaching assignment	STS‐survey, ProQOL	Other: This paper reports STS separately on one scale; however, also reports it as CF with another scale	K‐12 educators	180	Descriptive, correlation, regression	NS years service; SIG (Teaching in same building: *r* = −.266); SIG (Current assignment: *r* = −.250)	30
5	Beasley and Norris (2021)	US	Peer‐reviewed Journal	Demographics, SCARS	ProQOL	STS distinct variable/outcome	School counsellors	55	ANOVA, descriptive, non‐parametric (Mann–Whitney and Kurskal–Wallis)	SIG (Coordination: β = −.32); SIG (Other: β = .42) SIG (Title I: *r* = −.32, U = 153.5, *z* = −2.38); NS (age, race, years exp.)	31
6	Berger and Nott (2024)	Aus.	Peer‐reviewed Journal	Demographics, MH hx., trauma hx., exp. to student trauma, trauma training, knowledge and confidence	ProQOL	STS distinct variable/outcome	Teachers	302	Correlation, regression, descriptive	NS (age, gender, trauma training) SIG (direct/indirect student trauma; *r* = .13; β = .14) SIG (trauma hx: *r* = .16; β = .16); SIG correlation (mental health hx; *r* = .14) NS in regression	31
7	Borntrager et al. (2012)	US	Peer‐reviewed Journal	Social support, demographics	STSS, ProQOL	STS distinct variable/outcome	School staff	300	Regression, descriptive	SIG (Organization discouraging support‐seeking: β = 20.23) SIG (Intent to seek other employment: β = .26) NS (personal trauma hx)	25
8	Bozgeyikli (2018)	Turkey	Peer‐reviewed Journal	Psych. needs (success, relationship, autonomy, dominance)	ProQOL	Combined or reported as compassion fatigue (CF)	Spec. ed. teachers	238	Pearson correlation, regression	SIG (psych. need of Relationship: *r* = −.588, β = .457) others NS	20
9	Brady (2021)	US	Dissertation/Thesis	Locus of Control (LOC)	ProQOL	STS distinct variable/outcome	School counsellors	90	Multiple regression, moderation analysis, descriptive	SIG (Locus of Control: *r* = −.354)	35
10	Brown (2016)	US	Dissertation/Thesis	LSFT, SCNS	ProQOL	STS distinct variable/outcome	Preschool teachers	34	Descriptive, regression, ANOVAs	SIG (LSFT: *r* = .42); NS (Competence/Need for support)	35
11	Brown and Biddle (2023)	US	Peer‐reviewed Journal	Demographics, ACEs, school climate, resilience, empathy, CS	ProQOL	STS distinct variable/outcome	K‐12 teachers	542	SEM (structural modelling), correlation, regression	SIG (Perceived Student ACEs: *r* = .23) SIG (Personal trauma hx: *r* = .277) SIG (Resilience: *r* = −.316, −) SIG (CS: *r* = −.322) SIG (Org Support: *r* = −.14; Relational Support: *r* = −.10)	34
12	Buehler (2019)	US	Dissertation/Thesis	Grade level	ProQOL	Combined or reported as compassion fatigue (CF)	K‐8 educators	182	ANOVA	SIG (KG–2 vs. Third–5: diff = 3.03; KG–2 vs. Sixth–8: diff = 2.71); NS (third‐5 vs. sixth‐8)	30
13	Castro Schepers (2023)	US	Peer‐reviewed Journal	School & teacher demographics	TSTSS	STS distinct variable/outcome	K‐12 teachers	115	Regression, descriptive	SIG (Non‐White; B = −7.13) SIG (Working Class: B = 6.01; Upper Middle: B = −8.52) SIG (Elementary vs. High: B = −5.88) NS (gender, years exp.)	35
14	DeMatthews et al. (2019)	US	Peer‐reviewed Journal	Student/school demographics, principal demographics	ProQOL	STS distinct variable/outcome	Principals	86	Descriptive, correlation	SIG (Emotional exhaustion *r* = .590, Depersonalization *r* = .415, Personal accomplishment *r* = −.407) SIG (CS: *r* = −.361) NS (years exp.; school‐level poverty)	27
15	DeMatthews et al. (2023)	US	Peer‐reviewed Journal	Principal exp.	ProQOL	STS distinct variable/outcome	Principals	199	Correlation, descriptive, regression (BO as dependent)	NS (years exp.)	31
16	Denham (2019)	US	Dissertation/Thesis	School disrepair	STSS	STS distinct variable/outcome	Teachers	172	T‐Tests	SIG (non‐blighted; *t* = −6.34)	29
17	Erçevik (2019)	Turkey	Peer‐reviewed Journal	Problem‐solving style, demographics, trauma exp.	STSS	STS distinct variable/outcome	School psy. counsellors	268	Regression, correlation, independent t‐test	SIG (age‐older *r* = −.182) SIG (Seniority: *r* = −.134) SIG [borderline] (No Client trauma: *t* = − 1.95) SIG (No Trauma history: *t* = −3.22) SIG (NPO: *r* = .364, β = .34)	22
18	Farrell (2022)	US	Dissertation/Thesis	Work env., demographics, hope, resilience, self‐efficacy, CS	ProQOL	STS distinct variable/outcome	K‐12 teachers	341	Regression, correlation, ANOVA	SIG (female gender: *r* = .24, β = .143) SIG (Years in Education: β = −.126) SIG (Resilience: *r* = −.46, β = −.329) SIG (CS: *r* = −.65, β = −.256) NS (SES, School‐level poverty, self‐care/self‐efficacy)	29
19	Fute et al. (2022)	China	Peer‐reviewed Journal	Work engagement, gender, grade level	ProQOL	Other: STS reported both distinctly and as part of CF in different analysis	Teachers	3147	Correlation	SIG for CF (years exp.: *r* = −.377) SIG (Work Engagement [vigour, dedication, and absorption] *r* = .49, *r* = .34, and *r* = .32)	25
20	Glass (2022)	US	Dissertation/Thesis	Social–emotional competence, demographics	ProQOL	STS distinct variable/outcome	Pre‐service teachers	51	Regression, correlation, moderation	NS (socio‐emotional competence, demographics)	33
21	Gomez (2021)	US	Dissertation/Thesis	Teacher characteristics	ProQOL	STS distinct variable/outcome	Teachers	65	*t*‐tests	SIG (General education vs. other roles: t(63) = −2.144) NS (years exp., school‐level poverty)	27
22	Honsinger (2018)	US	Dissertation/Thesis	Exp. to student trauma, trauma hx., boundaries, school level, trauma training	VTS	Combined or reported as vicarious trauma (VT)	School counsellors	217	ANOVA, correlation/regression	SIG in correlation (50% trauma case; *r* = .14) NS in regression SIG (SODS/self‐differentiation; β = −.35) SIG (grade level) NS (trauma training, personal trauma hx)	38
23	Hydon (2016)	US	Dissertation/Thesis	Demographics, trauma exp.	ProQOL, STSS	STS distinct variable/outcome	Teachers	136	Correlation, regression, ANOVAs	SIG via ProQOL(CS: *r* = −.311) NS via STSS	31
24	Iacolino et al. (2023)	Italy	Peer‐reviewed Journal	Remote work risks, EI, metacognition, demographics	STSS	STS distinct variable/outcome	Teachers	604	*t*‐tests, ANOVA, correlation also mediation/moderation model analysis	SIG (Emotional intelligence: *r* = −.479, Metacognition: *r* = −.486) SIG (Remote work risk factors: *r* = .31)	27
25	Jackson (2024)	US	Dissertation/Thesis	Resilience, intent to leave, demographics	ProQOL	STS distinct variable/outcome	Teachers	529	Mann–Whitney, correlation, descriptive, regression	SIG (Female > Male: *U* = 17,452, *z* = −3.66) SIG (resilience: rs = −.359) SIG (CS: rs = −.394) SIG (Intent to Leave: rs = .32)	36
26	Katsigeorgis (2021)	US	Dissertation/Thesis	Coping, demographics, self‐care, trauma hx., absenteeism	ProQOL	STS distinct variable/outcome	School psy., counsellors, social workers	230	Correlation, regression, descriptive	SIG (Negative coping: *B* = .195) NS (age, years exp., personal mental/phys health)	34
27	Klemme (2023)	US	Dissertation/Thesis	SDH, trauma, work stressors, supports, demographics	STSS	STS distinct variable/outcome	School personnel	175	Correlation, regression, descriptive	SIG (younger β = −.17) SIG in 2 models (higher education β = .177) NS in final 2 SIG (elementary school: β = .163) SIG (Teachers: β = .269) SIG (Trauma: β = .313) SIG in model 1 (Childhood adversity: β = .22) SIG (Supervisor support: β = −.16); NS (gender, years exp., peer/personal support) SIG (Work hazards: β = .29)	38
28	Knox et al. (2023)	US	Peer‐reviewed Journal	ACEs hx., TIP efficacy, demographics	Summed score Adams et al. (2001)	STS distinct variable/outcome	School personnel	566	Descriptive, ANOVA, latent profile analysis, moderation analysis	SIG ANOVA latent profile (low ACES vs. Average vs. Neglected) F(3, 562) = 19.87; NS (Neglected vs. High ACES) NS moderation (trauma‐informed Self‐efficacy)	31
29	Lassri (2023)	Israel	Peer‐reviewed Journal	Demographics, mentalizing, emotion reg., self‐compassion, trauma hx., social support, etc.	ProQOL	STS distinct variable/outcome	Teachers, admin.	350	Descriptive, latent profile analysis, ANOVAs	SIG (less trauma training: Risk Group) SIG (Risk group profile ↑ childhood trauma) Bayesian analysis, group STS mean difference [less childhood trauma] = −3.14, *r* Cauchy = −.71 SIG (Risk group: ↓ self‐compassion, reappraisal, mentalizing; ↑ self‐criticism, suppression Resilience group opposite) SIG (Risk Group higher STS/lower CS, Resilience group opposite) SIG (Resilience group: ↑ social support; Risk group: ↓ support)	30
30	Levkovich and Ricon (2020)	Israel	Peer‐reviewed Journal	Demographics, optimism, perceived stress, psy. symptoms	ProQOL	STS distinct variable/outcome	School counsellors	139	Correlation, ANOVA, descriptive, mediation analysis	SIG (Elementary > High School: *F* = 3.539) SIG (*r* = −.34 for optimism; *r* = .40 for perceived stress; *r* = .59 for emotional distress) NS (age)	27
31	Lewis (2021)	US	Dissertation/Thesis	Demographics, EI, self‐advocacy	ProQOL	STS distinct variable/outcome	School counsellors	194	Correlation, ANOVA, regression	SIG (TEIQue: *r* = −.448; β = −.463) SIG (CS: *r* = −.363) SIG (SCSA: *r* = −.23) NS in regression	28
32	Monahan (2023)	US	Dissertation/Thesis	Trauma hx., self‐care, demographics, prof. support, dosage of trauma	STSS, ProQOL	STS distinct variable/outcome	K‐12 staff	225	Descriptive, Correlation, ANOVA, Regression, moderation	SIG [in professional set model] (Male β = −.15) NS (in personal set model) SIG (Black/AA; β = −.15) SIG [only in step 1] (Education – 2 year or less; β = −.15) SIG [only in step 1] (part of crisis team; β = .13) NS (in subsequent steps) SIG (perceived dosage β = .24) SIG (total trauma score; β = .28) SIG (self‐care β = −.26) SIG (supervisor support β = −.19) also moderates effect of perceived student trauma dosage. NS (age, years exp., peer support)	36
33	Neighbors (2023)	US	Dissertation/Thesis	Coping	STSS	STS distinct variable/outcome	School admin.	94	Correlation, descriptive, ANOVA	SIG (Avoidance Coping styles: e.g. disengagement *r* = .74; Substance Use *r* = .39; Emotion focused styles: e.g. Self‐Blame *r* = .45; Problem focused styles: e.g. Planning *r* = .32) NS (social support, advice seeking, religious practice) NS (gender, years exp.)	35
34	O'Toole and Dobutowitsch (2023)	Ireland	Peer‐reviewed Journal	Self‐compassion, ARTIC, demographics	ProQOL	STS distinct variable/outcome	Teachers, admin.	377	Correlation	SIG (Self‐compassion: *r* = −.376; ARTIC‐35: *r* = −.195) SIG (CS: *r* = −.341)	27
35	Ollison (2019)	US	Dissertation/Thesis	Demographics	ProQOL	STS distinct variable/outcome	Teachers	100	ANOVA, descriptive, regression	SIG [Non‐binary Teachers (Elementary, Correlation) *r* = .399] NS male/female SIG [Ethnicity: Asian/Pacific Islander (High School) *r* = .325] NS (prefer not to say) SIG [SED Status (Middle School) *r* = .509; B = 7.66, % African American Students (Middle School) *r* = .414; B = 6.22, % White Students (Middle School) *r* = −.648; B = −10.5] NS (years exp.)	35
36	Prout et al. (2023)	US	Peer‐reviewed Journal	Health, RAW, demographics	ProQOL	STS distinct variable/outcome	School staff	502	Correlation, ANOVA, latent profile analysis	SIG (Living Authentically: *r* = −.22; Finding Your Calling: *r* = −.20; Maintaining Perspective: *r* = −.48; Managing Stress: *r* = −.25; Interacting Cooperatively: *r* = −.12) NS (personal phys health)	26
37	Rankin (2022)	US	Dissertation/Thesis	Demographics, trauma hx.	ProQOL	STS distinct variable/outcome	Teachers	158	Descriptive, ANOVA, regression	SIG (Females > Males: t = 4.631) NS (years exp.) SIG (History of trauma: t = 5.293)	33
38	Rumsey et al. (2020)	US	Peer‐reviewed Journal	Demographics, trauma exp.	ProQOL	STS distinct variable/outcome	School counsellors	361	ANOVA, descriptive, regression	SIG (Years exp.: β = −.11) SIG (Title I status: *F*(1,354) = 11.96, *d* = .37) NS (grade level, caseload size) SIG (Secondary trauma: β = .22)	31
39	Shoieb (2020)	US	Dissertation/Thesis	Demographics	ProQOL	STS distinct variable/outcome	K‐8 teachers	132	Descriptive, t‐tests	SIG (Females > Males: *t* = 3.829; *d* = .94) NS (years exp., grade level)	33
40	Simon (2019)	US	Dissertation/Thesis	Demographics, ACEs, emotion regulation, teacher sensitivity, relationships	ProQOL	STS distinct variable/outcome	School staff	150	SEM (structural equation modelling; for latent profiles), correlation, regression	SIG (Black: β = −.24; Other non‐White: β = −.11) SIG (Student socio‐emotional difficulties: *r* = .26; student ACEs *r* = .22) SIG (teacher ACEs: β = .19) SIG (Cognitive Reappraisal: β = − .32; Compassion Resilience: *r* = −.51) NS (gender, relationship qual, sensitivity)	33
41	Steen (2019)	US	Dissertation/Thesis	Demographics	ProQOL	Other: The author conceptualizes STS as CF in their variable structure but reports STS separately in tables	Spec. & gen. ed. teachers	260	Correlation, descriptive, MANOVA	SIG (CS: *r* = −.25) SIG (Prof. Distress; *r* = .21) (NS other variables)	36
42	Steketee (2020)	US	Dissertation/Thesis	Opioid zones, demographics, ACEs perceptions	STSS	STS distinct variable/outcome	K‐12 teachers	450	Descriptive, correlation (spearman's rho)	NS (opioid zones, demographics)	35
43	Stiles (2020)	US	Dissertation/Thesis	Demographics, trauma exp., self‐efficacy, commitment, social support, org. health	STSS	STS distinct variable/outcome	K‐12 teachers	135	Descriptive, ANOVA	SIG (heard from student: β = .21; Total indirect exposure: β = .16; Indirect Trauma Exposure: Chronicity: *r* = .42) SIG (General teaching self‐efficacy: β = .21); NS (personal self‐efficacy) SIG (Social support within school: β = −.397) SIG (Teacher commitment: β = −.29)	32
44	Wells (2004)	US	Dissertation/Thesis	Demographics	ProQOL	Combined or reported as compassion fatigue (CF)	School counsellors	78	Descriptive, Correlation	NS (gender)	30
45	Williams (2023)	Jamaica	Dissertation/Thesis	Demographics, VT‐ORG	Stakeholder Survey	Combined or reported as vicarious trauma (VT)	HS stakeholders	108	MANOVA, ANOVA	SIG (Role η^2^ = .39) SIG (school η^2^ = .40)	35
46	Wilson (2020)	US	Dissertation/Thesis	STSI‐OA, demographics	ProQOL	STS distinct variable/outcome	HS counsellors & social workers	68	ANOVA, descriptive	SIG (social worker > counsellor t = 2.04) SIG (Supervisors attending to STS risks: β = −.49) NS (years exp.)	32
47	Woloshyn et al. (2023)	Canada	Peer‐reviewed Journal	Coping, resilience, stress, well‐being	STSS	STS distinct variable/outcome	Educators	115	Correlation, ANOVA, descriptive	NS (gender)	26
48	Yu et al. (2022)	China	Peer‐reviewed Journal	Demographics, school role	ProQOL	Other: STS is reported separately for ANOVA, but then combined with BO (as CF) for regression model results	Teachers	1527	ANOVA, descriptive, t‐tests	SIG (primary < secondary and secondary vocational; *F* = 9.540) SIG for CF (east < Midwest; t ≈ −2.02) NS (demographics) NS (title or role) NS (subject) [note: title/role SIG for CF]	31

Abbreviations: BO, burnout; CF, compassion fatigue; CS, compassion satisfaction; HS, high school; K‐8/K‐12, kindergarten to 8th/12th grade; NS, not significant; ProQOL, Professional Quality of Life scale; SIG, significant; STSS, Secondary Traumatic Stress Scale; TSTSS, Teacher Secondary Traumatic Stress Scale; VT, vicarious trauma; VTS, Vicarious Trauma Scale.

### Critical appraisal of sources

Since the synthesis of evidence would be primarily derived from a range of quantitative studies, including both peer‐reviewed and grey literature, we deemed it appropriate to conduct a systematic appraisal of the individual sources, which aligns with PRISMA‐ScR (Tricco et al., 2018). Two independent reviewers conducted the appraisal of sources utilizing the Quality Assessment with Diverse Studies (QuADS; Harrison et al., 2021) tool. The QuADS uses 13 criteria to appraise methodological and reporting quality and is an updated version of the Quality Assessment Tool for Studies with Diverse Designs (QATSDD) and was selected for its ability to appraise diverse quantitative analyses, including cross‐sectional and longitudinal (Harrison et al., 2021). Both independent reviewers followed the six‐step protocol outlined in the QuADS user guide. All QuADS criteria and protocol documents are available in the Figures S2 and S3.

### Data analysis and reporting

Given the state of the literature, with its wide range of variables and inconsistent analytical methods, a scoping review was most appropriate. We used narrative synthesis, figures, and tables (tabular methods) to report the findings and heterogeneity in studies. Reporting was informed by the Synthesis Without Meta‐Analysis (SWiM) guideline, which is designed to complement PRISMA (Campbell et al., 2020). We enumerated the significant findings and narratively reported the effect sizes, where possible. Quantitative information on explanatory variables (including statistical significance, effect direction, and effect size, where available) was summarized and tabulated to outline areas of consensus, contradictory findings, or an absence of evidence, thereby concisely mapping and synthesizing the existing literature for the reader. Where statistical significance was reported, it followed the *p* < .05 standard for significance (Hanna & Dempster, 2013), unless otherwise noted, such as a Bonferroni adjustment. Where effect sizes or magnitudes were narratively reported, small/modest, medium/moderate, and large were based on each value's general guidelines or benchmarks proposed by Cohen (1988).

## RESULTS

The search of five preregistered databases identified 1238 records. After deduplication and title and abstract screening, 120 records met eligibility for full‐text screening. One record could not be retrieved due to being embargoed, leaving 119 records available for retrieval and full‐text assessment. Ultimately, 48 studies met all inclusion criteria and were included in the synthesis. Figure 1 illustrates the full PRISMA flow diagram.

### Included studies characteristics

For clarity in reporting the results, all included studies were assigned a number from 1 to 48 and can be identified according to their order in Table 1. Sample sizes across the 48 studies ranged from 34 participants (10) to 3147 participants (19), with a mean sample size of 315.02 (*SD* = 491.29). Most studies (37; 77%) were conducted in the United States (USA). Three countries (China, Israel, and Turkey) had two studies each. Australia, Canada, Ireland, Italy, and Jamaica each had a single study represented. Regarding research years, the oldest study was from 2004 (44), and the most recent was from 2024 (2), with most conducted within the 2020s. Following the preregistered inclusion criteria, all 48 studies obtained samples from a school staff population below the tertiary level. Twenty‐nine of the included 48 studies were doctoral theses/dissertations. All included studies were cross‐sectional designs except one (11), which included both longitudinal data (over 9 months) and cross‐sectional data. Detailed characteristics of each study are presented in Table 1.

### Critical appraisal of sources

Two independent reviewers appraised each study using the Quality Assessment for Diverse Studies (QuADS; Harrison et al., 2021). Based on 13 criteria items, the lowest possible score was 0, and the highest possible score for a study was 39. As shown in Table 1, Study 8 received the lowest rating of 20, and Studies 25 and 27 received the highest scores of 38 each. The mean rating across studies was 31.02 (SD = 4.03). Inter‐rater reliability was calculated using intra‐class correlation and was excellent, ICC (3,1) = .98, 95% CI [.98, .99], *F*(623, 623) = 58.63, *p* < .001 (Koo & Li, 2016). All studies received the highest possible rating of ‘3’ on QuADS items 4 (appropriate study design), 7 (format and content of data collection tool), and 11 (method of analysis was appropriate), which reflect a suitable level of quantitative design and data collection quality across studies. Lower‐rated studies (e.g., 3 and 7), although being rated high in the above items, received ratings of ‘1’ or ‘0’ on items dealing with statements of theoretical underpinning, clear research aim statements, and details on recruitment. Many studies (e.g., 1–4, 6–9, 14–21) received ‘0’ ratings on item 12 (evidence that research stakeholders were considered in design), indicating that these studies did not make clear statements about their collaborations or considerations for the intended populations, such as integrating feedback from a pilot study. This suggests that while the quantitative methods were rated highly across all studies, some studies did not sufficiently outline their theoretical foundation and aims, and did not explicitly show their stakeholder considerations, as defined by the QuADS criteria. Nevertheless, as evidenced by a mean score of 31 out of a possible 39, the included studies were generally of adequate quality and showed good quantitative data collection and analysis methodology.

### Explanatory variables

To map and synthesize the wide range of variables across the 48 studies, we categorized the explanatory variables based on the refined trauma model by MacRitchie and Leibowitz (2010), which states that STS‐moderating variables are grouped by individual characteristics (e.g., age, gender), personal variables (e.g., prior trauma), and environmental context (e.g., social support, school environment). The six explanatory (‘predictor’) categories are: *1. Biological/Personal Characteristics* (e.g., age, gender), *2. Professional Demographics* (e.g., years of experience, role), 3. *Exposure to Student Trauma/Personal Trauma‐Related Variables* (e.g., trauma history, student ACEs), 4. *Cognitive and Emotional Resources and Coping* (e.g., problem‐solving style, emotional intelligence), 5. *Professional/Work Support and Social Support* (e.g., supervisor support, organization climate), and 6. *Personal Professional Qualities* (e.g., time management, discipline). Table 2 summarizes variables within each domain, and Table 3 visually maps the findings from each study.

**TABLE 2:** Predictor/Variable grouping summary.

Predictor category	Predictor variables (IV across studies)
1. Biological/Personal Demographics	Age, Gender, Race/Ethnicity, Socioeconomic Status, Education Level
2. Professional Demographics	Grade level taught; teaching assignment, Years of service/experience, Teaching tenure, Teacher position, Level of Degree, Beginning Teacher, Experienced Teacher, Current teaching assignment, Teaching in same building, Teacher Role, Novice vs. Experienced, Education, Years Employed
	Caseload size, Counselling‐related activities (Counselling, Consultation, Coordination, Curriculum, Other), Grade level taught, Number of Identified Students with Specialized Needs, Number of Instruction Transitions, Teacher Role, Campus Role, Teaching assignment (e.g., middle & high school choir vs. single level), Content (general vs. special education), Working in single vs. multiple buildings, Degree Type
	School type (Elementary, Middle, High), Title I qualification, School condition (blighted/disrepair vs. non‐blighted), School Demographics, Campus Location, Opioid epidemic zone, Region, School setting factors, % Economically disadvantaged students, SED status, student racial composition (% White and % non‐White)
	Proportion of trauma‐exposed students on caseload (including perceived)
3. Exposure to Student Trauma/Personal Trauma‐Related Variables	Secondary traumatic stress exposure, Trauma contact, Exposure to student trauma, Perceived Dosage of Student Trauma, Student Trauma categories, Client Traumatic Experience, Indirect trauma exposure, Perceived Student ACEs
	Personal trauma history; ACEs
	Personal mental/Physical health history
4. Cognitive and Emotional Resources & Coping	Emotional Regulation, Resilience, Hope–Agency, Hope–Pathway, Self‐Care, Negative coping, Planning, Self‐blaming, Humour, Behavioural Disengagement, Venting, Denial, Mental Disengagement, Substance Use, Social Problem‐Solving Dimensions: PPO, NPO, RPS, ICPS, APS, Living Authentically, Finding Your Calling, Maintaining Perspective, Managing Stress, Interacting Cooperatively
	Intrapersonal Mindfulness, Interpersonal Mindfulness, Locus of Control, Cognitive Reappraisal, Metacognition, Emotional intelligence, Self‐other differentiation (SODS_Score), Trait Emotional Intelligence (TEIQue), Perceived knowledge and confidence, Optimism, ARTIC‐35 (attitudes towards trauma‐informed care). Psychological needs domains (Success, Relationship, Dominance, Intrapersonal), Compassion satisfaction
5. Professional/Work Support and Social Support	Organization encourages social support‐seeking behaviours, Supervisor support, Colleague Support, District Environment, Emotional connection with co‐workers. social support at school, organizational support/relational support
6. Personal Professional Qualities	Time management, discipline, professional investment, Loss of satisfaction in Teaching (LSFT), intention to leave, teacher commitment

**TABLE 3:** STS predictor summary.

	1.				2.						3.			4.				5.	6.
Study #	Age	Gender	Race / Ethnicity	SES & Education	Years Teaching / Experience	Grade Level / Teaching Assignment	Role / Duty / Title	School‐Level Poverty Indicator	Caseload Size / % Trauma‐Exposed Students	School Building Disrepair	Direct / Indirect Student Trauma Exposure	Personal Trauma History / ACEs	Personal Mental / Physical Health History	Mindfulness / Coping / Reappraisal (TEI)	Compassion Satisfaction	Psych. Needs	Self‐Care, Self‐Advocacy & Self‐Efficacy	Supervisor & Leadership Support	Time Mgmt, Discipline & Commitment
**1**	‐	‐	‐	‐	‐	‐	‐	‐	‐	‐	**✓**	‐	‐	‐	‐	‐	‐	**✓**	**✓**
**2**	‐	‐	‐	‐	‐	‐	‐	‐	**✓**	‐	**X**	‐	‐	‐	**X**	‐	‐	‐	‐
**3**	‐	‐	‐	‐	‐	‐	‐	‐	‐	‐	‐	‐	‐	**✓**	‐	‐	‐	‐	‐
**4**	‐	‐	‐	‐	**X**	**✓**	‐	‐	‐	‐	‐	‐	‐	‐	‐	‐	‐	‐	‐
**5**	**X**	‐	**X**	‐	**X**	‐	**✓**	**✓**	‐	‐	‐	‐	‐	‐	‐	‐	‐	‐	‐
**6**	**X**	**X**	‐	**X**	**X**	‐	‐	‐	‐	‐	**✓**	**✓**	**✓**	‐	‐	‐	‐	‐	‐
**7**	‐	‐	‐	‐	‐	‐	‐	‐	‐	‐	‐	**X**	‐	‐	‐	**X**	‐	**✓**	**✓**
**8**	‐	‐	‐	‐	‐	‐	‐	‐	‐	‐	‐	‐	‐	‐	‐	**✓**	‐	‐	‐
**9**	‐	‐	‐	‐	‐	‐	‐	‐	‐	‐	‐	‐	‐	**✓**	‐	‐	‐	‐	‐
**10**	‐	‐	‐	‐	‐	‐	‐	‐	‐	‐	‐	‐	‐	‐	‐	**X**	‐	‐	**✓**
**11**	‐	‐	‐	‐	‐	‐	‐	‐	‐	‐	**✓**	**✓**	‐	**✓**	**✓**	‐	‐	**✓**	‐
**12**	‐	‐	‐	‐	‐	**✓**	‐	‐	‐	‐	‐	‐	‐	‐	‐	‐	‐	‐	‐
**13**	‐	**X**	**✓**	**✓**	**X**	**✓**	‐	‐	‐	‐	‐	‐	‐	‐	‐	‐	‐	‐	‐
**14**	‐	‐	‐	‐	**X**	‐	‐	**X**	‐	‐	‐	‐	‐	**✓**	**✓**	‐	‐	‐	‐
**15**	‐	‐	‐	‐	**X**	‐	‐	‐	‐	‐	‐	‐	‐	‐	‐	‐	‐	‐	‐
**16**	‐	‐	‐	‐	‐	‐	‐	‐	‐	**✓**	‐	‐	‐	‐	‐	‐	‐	‐	‐
**17**	**✓**	‐	‐	‐	**✓**	‐	‐	‐	**✓**	‐	‐	**✓**	‐	**✓**	‐	‐	‐	‐	‐
**18**	‐	**✓**	‐	**X**	**✓**	**X**	‐	**X**	‐	‐	‐	‐	‐	**✓**	**✓**	‐	**X**	‐	‐
**19**	‐	**X**	‐	‐	**✓**	**X**	‐	‐	‐	‐	‐	‐	‐	‐	‐	‐	‐	‐	**✓**
**20**	‐	‐	‐	**X**	‐	‐	‐	‐	‐	‐	‐	‐	‐	**X**	‐	‐	‐	‐	‐
**21**	‐	‐	‐	‐	**X**	‐	**✓**	**X**	‐	‐	‐	‐	‐	‐	‐	‐	‐	‐	‐
**22**	‐	‐	‐	**X**	‐	**✓**	‐	‐	**✓**	‐	‐	**X**	‐	**✓**	‐	‐	‐	‐	‐
**23**	‐	‐	‐	‐	‐	‐	‐	‐	‐	‐	‐	‐	‐	**✓**	‐	‐	‐	‐	‐
**24**	‐	‐	‐	‐	‐	‐	‐	‐	‐	‐	‐	‐	‐	**✓**	‐	‐	‐	‐	**✓**
**25**	‐	**✓**	‐	‐	‐	‐	‐	‐	‐	‐	‐	‐	‐	‐	**✓**	‐	‐	‐	**✓**
**26**	**X**	‐	‐	‐	**X**	‐	‐	‐	‐	‐	‐	‐	**X**	**✓**	‐	‐	‐	‐	‐
**27**	**✓**	**X**	‐	**✓**	**X**	**✓**	**✓**	‐	‐	‐	**✓**	**✓**	‐	‐	‐	‐	‐	**✓**	**✓**
**28**	‐	‐	‐	‐	‐	‐	‐	‐	‐	‐	‐	**✓**	‐	‐	‐	‐	**X**	‐	‐
**29**	‐	‐	‐	**✓**	‐	‐	‐	‐	‐	‐	‐	**✓**	‐	**✓**	**✓**	‐	‐	**✓**	‐
**30**	**X**	‐	‐	‐	‐	**✓**	‐	‐	‐	‐	‐	‐	‐	**✓**	‐	‐	‐	‐	‐
**31**	‐	‐	‐	‐	‐	‐	‐	‐	‐	‐	‐	‐	‐	**✓**	**✓**	‐	**✓**	‐	‐
**32**	**X**	**✓**	**✓**	**✓**	**X**	‐	**✓**	‐	‐	‐	**✓**	**✓**	‐	‐	‐	‐	**✓**	**✓**	‐
**33**	‐	**X**	‐	‐	**X**	‐	‐	‐	‐	‐	‐	‐	‐	**✓**	‐	‐	‐	**X**	‐
**34**	‐	‐	‐	‐	‐	‐	‐	‐	‐	‐	‐	‐	‐	**✓**	**✓**	‐	‐	‐	‐
**35**	‐	**✓**	**✓**	‐	**X**	‐	‐	**✓**	‐	‐	‐	‐	‐	‐	‐	‐	‐	‐	‐
**36**	‐	‐	‐	‐	‐	‐	‐	‐	‐	‐	‐	‐	**X**	**✓**	‐	‐	‐	‐	‐
**37**	‐	**✓**	‐	‐	**X**	‐	‐	‐	‐	‐	‐	**✓**	‐	‐	‐	‐	‐	‐	‐
**38**	‐	‐	‐	‐	**✓**	**X**	‐	**✓**	**X**	‐	**✓**	‐	‐	‐	‐	‐	‐	‐	‐
**39**	‐	**✓**	‐	‐	**X**	**X**	‐	‐	‐	‐	‐	‐	‐	‐	‐	‐	‐	‐	‐
**40**	‐	**X**	**✓**	‐	‐	‐	‐	‐	‐	‐	**✓**	**✓**	‐	**✓**	‐	‐	‐	‐	**X**
**41**	**X**	**X**	**X**	‐	‐	‐	‐	‐	‐	‐	‐	‐	‐	‐	‐	‐	‐	‐	‐
**42**	‐	**X**	‐	‐	**X**	‐	**X**	**X**	‐	‐	‐	‐	‐	‐	**✓**	‐	‐	‐	**✓**
**43**	**X**	**X**	**X**	‐	‐	**X**	‐	**X**	‐	‐	**✓**	‐	‐	**X**	‐	‐	**✓**	**✓**	**✓**
**44**	‐	**X**	‐	‐	‐	‐	‐	‐	‐	‐	‐	‐	‐	‐	‐	‐	‐	‐	‐
**45**	‐	‐	‐	‐	‐	‐	**✓**	**✓**	‐	‐	‐	‐	‐	‐	‐	‐	‐	‐	‐
**46**	‐	‐	‐	‐	**X**	‐	**✓**	**X**	‐	‐	‐	‐	‐	‐	‐	‐	‐	**✓**	‐
**47**	‐	**X**	‐	‐	‐	‐	‐	‐	‐	‐	‐	‐	‐	‐	‐	‐	‐	‐	‐
**48**	‐	**X**	‐	**X**	**X**	**✓**	**X**	**✓**	‐	‐	‐	‐	‐	‐	‐	‐	‐	‐	‐

*Note*: (✓) Sig; (X) NS; (−) Not investigated.

**Full variable list by predictor category:** **1. Biological/Personal Demographics** Age; Gender; Race/Ethnicity; Socioeconomic Status and Education. **2. Professional Demographics** Years teaching/Experience; Grade level taught/Teaching Assignment; Role/Duty/Title; School‐Level Poverty indicator (e.g., Title I status, %African American, free and reduced lunch); Caseload size or Proportion of trauma‐exposed students on caseload; School building Disrepair. **3. Exposure to Student Trauma / Personal Trauma‐Related Variables** Direct and indirect student trauma exposure; Personal trauma history (e.g., ACEs); Personal mental/physical health history. **4. Cognitive and Emotional Resources & Coping** Intrapersonal mindfulness, resilience, negative problem solving, cognitive reappraisal, trait emotional intelligence (TEI), self‐compassion, self‐differentiation, Locus of Control; Compassion satisfaction; Psychological needs (Success, Relationship, Dominance, Autonomy); Self‐care, Self‐advocacy and Self‐Efficacy. **5. Professional/Leadership Support & Social Support** Supervisor support; Trauma‐informed leadership practices; organizations that encourage support seeking; social support at school; organizational support/relational support. **6. Personal Professional Qualities** Time management, discipline, professional investment, Loss of Satisfaction in Teaching (LSFT), teacher commitment, Intent to Leave.

#### Biological/personal demographics

Findings for this domain were largely inconsistent. Although many studies reported collecting personal demographic data, only a small subset examined the effects of these variables or compared them with STS. Specifically, 18 studies explicitly examined gender as a predictor, with only 33% (six; 18, 25, 32, 35, 37, 39) finding statistical significance. All except one (35) of these studies found that female gender was associated with higher STS, with a medium effect size. Study 35 found a significant medium association between non‐binary gender identification and higher STS. Similarly, age was a widely collected demographic data point, though minimally utilized as an explanatory variable. Only two of nine studies (22%) that explicitly investigated the effect of age found a significant effect. Studies 17 and 27 found that older age was significantly negatively associated with higher STS levels, with small and medium effects, respectively. For Race/Ethnicity, four studies (13, 32, 35, 40) out of seven (57%) found a significant effect. All except one (35) found that non‐White ethnicities (Black and Other non‐White) had significantly lower STS. Study 35 found that being of Asian/Pacific Islander ethnicity working at the high school level was moderately associated with higher STS. Findings for socioeconomic status (SES) or education level were mixed and did not show a clear pattern. Four (13, 27, 29, 32) of the nine studies (44%) investigating staff's SES or education level (including level of trauma training) as predictors of STS found significant medium effects in at least one regression step or model. Combined, these four studies reported that higher levels of education and working‐class SES were associated with higher STS, whereas lower education (2‐year degree or less) and upper‐middle‐class status were moderately negatively associated with STS. Among the three studies looking at trauma training (6, 22, 29), only Study 29 found a significant positive association between less trauma training and STS, with the others finding no significant effect.

#### Professional demographics

The evidence in this domain was mixed. Among the 21 studies that examined years of experience as a predictor of STS in school staff, only 19% (four; 17, 18, 19, 38) found statistically significant associations. All four showed small‐to‐medium negative associations between more experience and STS. Twenty studies examined two subcategories of variables involving teaching role/duty, grade level taught, and other assignment differences, yielding inconsistent findings. Four studies (12, 13, 22, 48) (20%) found significant small‐to‐medium group differences, in which lower grades were associated with lower STS. However, others (27, 30) (10%) found that lower grades were modestly associated with higher STS, with the rest finding no significant effect. Study 4 did not investigate grade level but examined a similar assignment difference and found a small negative association between teaching in the same building (typical for lower grades) and STS. Six studies (5, 21, 27, 32, 45, 46) (30%) found significant small‐to‐medium group differences in STS mean by staff role, duty, or title. Findings from studies 5, 21, 32, and 46 suggest that specific roles, such as school social workers, being part of a crisis team, and doing ‘other’ counselling duties outside of student coordination, are modestly associated with higher STS.

Continuing the Professional Demographics domain, 15 studies investigated two subcategories of variables related to school‐level socioeconomic indicators of poverty and the proportion of trauma‐exposed students. Across the 11 studies that evaluated the association of poverty indicators (e.g., Title I status, % African American students, opioid zone) on STS among school staff, 45% (five; 5, 35, 38, 45, 48) found significant medium‐to‐large effects. All five studies point to a higher rate of poverty status indicators, or serving a poorer area, being associated with elevated STS in staff. Furthermore, one of these five studies (35) indicates that having a higher percentage of White students versus Black (African American) students at the middle school level has a large negative association with STS. Important context for interpreting this finding is provided in the discussion section.

Of the four studies that explicitly investigated the proportion of trauma‐exposed students as a predictor of STS, 75% (three; 2, 17, 22) found significant medium effects, with more trauma‐exposed students being associated with higher STS. Concluding the Professional Demographics category is the variable ‘school building disrepair,’ which was examined in only one study (16). Yet, the blighted group was found to be significantly positively associated with higher STS without reporting an effect size.

#### Exposure to student trauma/personal trauma‐related variables

This category showed a more consistent pattern in the body of evidence for risk. Nine studies explicitly investigated perceived direct or indirect trauma exposure from students, and eight (89%) found that a higher proportion or intensity of student trauma exposure significantly relates to elevated STS. Findings from the eight studies (1, 6, 11, 27, 32, 38, 40, 43) indicate that school staff with a higher number of real or perceived trauma‐exposed students (e.g., ACEs) are at a greater risk of STS, with medium‐to‐large effects.

Across the 11 studies that examined staff's personal trauma history or ACEs as a predictor of STS, nine studies (6, 11, 17, 27, 28, 29, 32, 37, 40) found statistically significant positive relationships. In other words, 82% of studies in this category reported that higher levels of personal trauma or adverse childhood experiences are associated with higher levels of STS, with medium effect sizes. Personal mental health history and physical health were investigated in only three studies, of which only one (6) found a moderate, significant correlation with STS.

#### Cognitive and emotional resources and coping

This category presented a large sample of studies with some consistent patterns of evidence across many subcategories of variables. Over 30 studies explicitly investigated one or a combination of resilience domains, emotional intelligence, mindfulness, cognitive reappraisal, compassion satisfaction, psychological needs, or self‐care and advocacy‐related variables. Seventeen out of 19 (89%) studies investigating cognitive and emotional resources as predictors of STS found statistically significant relationships. Studies 3, 9, 11, 14, 17, 18, 22, 24, 25, 26, 29, 30, 31, 33, 34, 36, and 40 all found that higher use or presence of aspects of positive problem‐solving, emotional intelligence, resilience, mindfulness, reappraisal, self‐differentiation, and self‐compassion were negatively associated with STS. Conversely, maladaptive coping styles such as avoidance coping, negative problem‐solving orientation, and external locus of control were associated with significantly higher STS levels. These studies all reported medium‐to‐large effect sizes. Compassion satisfaction was investigated as a correlate of STS in 10 studies, of which 90% (nine; 11, 14, 18, 23, 25, 29, 31, 34, 42) found significant medium‐to‐large negative effects. Psychological Needs was another subcategory of variables investigated in this broader domain, with only one study (8) of three finding significance. This study found that only the need for relationship was negatively associated with STS, with a large effect size.

Five studies explicitly investigated self‐care, self‐advocacy, or self‐efficacy's impact on STS, with three (60%) finding significant results. Study 32 found self‐care to be significantly negatively correlated with STS, with a medium effect size. Similarly, Study 31 found that higher school counsellor self‐advocacy was associated with lower STS through correlation with a medium effect, though it did not make a significant contribution in regression analysis. Likewise, teacher and trauma‐informed self‐efficacy presented mixed findings. Study 43 found General Teaching Self‐efficacy (beliefs about teachers' efficacy in general) to positively predict STS in a regression model with a large effect, while Personal Teaching Self‐efficacy (beliefs about one's own teaching efficacy) was not significant. Conversely, Study 18, while finding a significant relationship between self‐efficacy and compassion satisfaction, did not find a direct significant correlation with STS (Bonferroni adjustment significance level).

#### Professional/leadership support and social support

There were also consistent patterns of evidence within some variables in this domain. Variables surrounding organizational support, social support at school, and trauma‐informed leadership were investigated by nine studies, of which eight (1, 7, 11, 27, 29, 32, 43, 46) (89%) found statistically significant associations. All eight consistently found that either higher organizational support, relational support, leadership/supervisor support, or leadership that encourages support‐seeking and attends to trauma risks were negatively associated with STS in school personnel, with medium‐to‐large effects. Social support (outside of school) had mixed findings, with two (1, 29) of four studies finding a significant negative moderate relationship.

#### Personal professional qualities

Variables in this category were less consistently conceptualized than others, making it difficult to synthesize meaningful patterns of evidence. However, personal professional qualities, such as time management stressors, discipline and motivation stressors, commitment, intent to leave, and loss of satisfaction in teaching (LSFT), were examined across 10 studies. Among these, nine (1, 7, 10, 19, 24, 25, 27, 41, 43) (90%) found significant associations between these qualities and STS, albeit with variability in constructs used, effect sizes, and time points (longitudinal). Generally, greater difficulties with time management, discipline and motivation, and work risk/hazards factors were moderately positively associated with STS across studies 1, 24, and 27. Teacher commitment and engagement (‘absorption or dedication’) had mixed findings from studies 19 and 43: the former found a medium positive association, whereas the latter found a medium negative association with teacher commitment and STS. The other studies examining loss of satisfaction from teaching, intent to leave, and professional distress found medium‐to‐large positive associations between these variables and STS.

### STS instruments

The Professional Quality of Life scale (ProQOL; Stamm, 2010) was the most commonly used instrument, appearing in 36 of the 48 studies (75%). The second most frequently used instrument was the Secondary Traumatic Stress Scale (STSS; Bride et al., 2004), which was used in 11 studies (23%). Some studies used both ProQOL and STSS in combination to measure different aspects of secondary trauma. Three studies (4, 13, 45) incorporated measures developed to assess STS in the school context. These measures were all versions of the ‘Teacher Secondary Traumatic Stress Scale’ (TSTSS; Castro Schepers & Young, 2022). Only one study (22) utilized a measure developed for vicarious trauma (VTS; Vrklevski & Franklin, 2008).

## DISCUSSION

The current literature on STS in schools is rapidly expanding but remains methodologically fragmented. Studies on risk and resilience factors for STS are characterized by a heavy reliance on cross‐sectional data and a lack of school‐specific measurement tools. Most studies borrow instruments (e.g., ProQOL) from clinical nursing and health or social work contexts, which may not fully capture the unique ecological demands of the school environment.

This scoping review charted and synthesized 48 quantitative studies to identify factors associated with risk and resilience for STS among school personnel. Our analysis reveals clear patterns of association in some domains and significant inconsistency in others. Although patterns in the evidence exist, no causal inferences can be made due to the cross‐sectional nature of the studies and variability of samples. Thus, while this review adds vital insight and synthesis to the current literature, we caution against overextrapolation of the findings.

Exposure to student trauma emerged as among the most robust risk factors identified in this review, with moderate‐to‐large effect sizes reported across 89% of the investigating studies (see Table 3). Notably, this variable included perceived dosage (staff's own interpretations and ratings of students' adverse stories; e.g., Study 32), suggesting that subjective appraisal of student trauma may form part of the secondary exposure picture. Personal trauma history was also consistently evidenced as a potential risk factor for elevated STS across 81% of studies exploring this variable (see Table 3), indicating that school personnel's own trauma experiences, including adverse childhood experiences, might increase vulnerability to STS in some contexts. Studies investigating STS in other professions have found similar associations with childhood trauma (Butler et al., 2018; Lacey, 2024; Tannenbaum, 2023), which lends support for the current synthesis.

The cognitive and emotional resources category showed some of the clearest evidence of protection in the review. Across 89% of the 19 studies examining these variables (see Table 3), higher levels of resilience, cognitive reappraisal, metacognition, emotional intelligence, and self‐compassion were associated with reduced STS in the current population. Conversely, maladaptive coping approaches, such as avoidance coping, which involves denying, minimizing, or not processing the stressful demands (Holahan et al., 2005), and a negative problem‐solving orientation, which involves a pessimistic outlook and perceived self‐inefficacy in problem‐solving ability (Robichaud & Dugas, 2005), were substantial risk factors. This pattern was distinguished by both its consistency and the magnitude of effects reported (medium to large). Furthermore, avoidance coping has been linked with generating and promoting new stressors, which might affect work relationships and compound risk over time (Holahan et al., 2005). Together, these findings position the cultivation of adaptive cognitive and emotional resources as potentially the most modifiable individual‐level protective mechanism available to school staff.

Compassion Satisfaction appears to be a significant protective factor, as evidenced by 90% of the 10 studies that investigated this construct (see Table 3). This indicates that a greater sense of fulfilment and satisfaction from serving students is strongly associated with lower STS. However, it should be noted that compassion satisfaction was measured using the same instrument as STS (ProQOL) in most studies (see Table 1), which was developed based on the compassion fatigue–compassion satisfaction model, where STS and compassion satisfaction are opposite ends of the Professional Quality of Life model (Stamm, 2010). Therefore, there is potential for psychometric overlap, given that the two constructs are measured using the same instrument across the included studies. This may explain the consistency and magnitude of the relationships found.

The role of psychological needs, self‐care, self‐efficacy, and self‐advocacy in influencing STS risk is less clear, based on the limited evidence in this review. Consequently, whether psychological needs and these factors are explanatory variables for STS in this population requires further research to reach a consensus. However, given the robust findings for cognitive and emotional resources and compassion satisfaction, these domains appear promising targets for continued research and the potential development of targeted interventions to reduce STS among school staff.

At the organizational level, professional and leadership support was shown to be perhaps the most consistent, policy‐actionable factor in this review, as 89% of the large sample of studies in this category reported significant negative associations with medium‐to‐large effect sizes (see Table 3). These findings position STS as a systemic organizational issue, rather than a purely individual one, indicating that outcomes are highly sensitive to institutional policy and climate. Specifically, robust organizational, relational, and supervisor support, particularly leadership that proactively addresses trauma risks and encourages support‐seeking, acts as a protective explanatory factor. Trauma‐informed models in education have demonstrated some relative success in implementation (Southall et al., 2023). Consequently, shaping leadership training and culture around these policies and practices (e.g., SAMHSA, 2014) may represent a systems‐level approach to STS mitigation that complements individually focused strategies.

The literature in the current review does not support gender or age as predictors of STS. While findings from studies across a range of professions suggest that women are often at greater risk for secondary traumatization (Baum et al., 2014) and similar trauma and stress‐related conditions like PTSD (Olff, 2017), females constituted over 75% of the samples across the included studies, which may affect the statistical power of sex‐specific outcomes. Future investigations should aim for a balanced sample when exploring gender‐ and sex‐specific outcomes. However, given the substantial representation of females in primary and secondary school staff in the regions with extensive literature on the current topic, ranging from 77% to 82% female (Australian Bureau of Statistics, 2022; Department for Education, 2023; National Center for Education Statistics, 2022), it is unlikely that the profession, as it stands today, would benefit from sex‐specific STS interventions. Instead, all sexes may benefit from interventions that recognize that a significant part of their population is at risk.

Race and ethnicity produced one of the more counterintuitive and complex patterns of the review. Across seven studies, only 57% found significant effects (see Table 3). All but one of these showed that identifying as a non‐White race/ethnicity associates with lower STS, which provides some evidence of a protective effect. This may appear initially counterintuitive, as people of colour tend to experience more trauma (Matheson et al., 2019) and have worse health outcomes (Seng et al., 2012). Explanations for the link between White staff and elevated STS risk include cultural disconnects and a ‘white saviour’ complex, wherein staff may be socially predisposed to view themselves as saviours for non‐White students (Aronson, 2017; Castro Schepers, 2023). This identity may add a layer of stress for White teachers when interacting with students from diverse backgrounds. Authors like Castro Schepers (2023) also acknowledge that, due to their experiences within systems of structural inequality and oppression, teachers of colour may reframe the issue as a system in need of fixing rather than a student, thereby creating some detachment from the individual. Nevertheless, due to inconsistent effect direction and mixed findings, there may not be sufficient evidence in the current study to conclude that race or ethnicity is an explanatory factor for STS. Moreover, since personal demographic variables such as age and race are not malleable, a holistic view of these factors should be employed. Similarly, due to the limited number of studies examining each variable and their inconsistent findings, there was insufficient evidence to categorically determine whether staff's SES, education level, or amount of trauma training significantly impacted STS.

The pattern of professional demographic variables, such as years of experience, grade level, and role assignment, was largely inconsistent across the literature. Only four of the 21 studies examining experience (19%; see Table 3) found significant effects, each linking greater experience with lower STS. The current analysis suggests that years of experience, by itself, is not necessarily an explanatory factor. The influence of confounding variables, such as resilience or coping strategies that staff may develop over time, could play a role and relate to more years of experience. Findings regarding teaching role/duty, grade level taught, and other assignment differences were inconsistent across 20 studies. Due to the variability in the conceptualization of role, duty, and assignment across studies, there is insufficient evidence to suggest whether these professional demographic variables are explanatory factors of STS. However, four studies investigating specific roles, such as school social workers, crisis team members, and those with counselling duties beyond student coordination, found these roles to be modestly associated with higher STS. This highlights a possible connection to the ‘Exposure to Student Trauma’ domain and the need for targeted support and preventative strategies for roles that are more exposed to students' trauma. Furthermore, existing literature supports that school staff in roles exposed to more traumatic material from others face a significant risk of STS (Ludick & Figley, 2017; Woodhams & Duran, 2024). Future research should strive to consistently group grade levels and include samples from a wider range of school contexts.

Evidence regarding the school's socioeconomic context and staff STS exposure remains inconsistent across the literature. Five studies found that Title I status (a poverty indicator in the United States) and other socioeconomic determinants (SED) of poverty, such as the percentage of ethnic minority students, were associated with higher STS levels. The finding that this was reflected in only 45% of examining studies (see Table 3) is likely explained by variation in how poverty was operationalized; as studies reporting non‐significant results tended to use broader poverty proxies, such as school region (e.g., Study 48) or opioid epidemic zones (e.g., Study 42), rather than direct poverty SEDs. Given these inconsistencies, there is insufficient evidence to position school‐level poverty as an explanatory factor for STS. It is more plausibly a correlate of higher trauma exposure within the student body, and future research should strive for consistent operationalization of this type of indicator.

We believe it is important to note that the percentage of African American or White students functions as a poverty indicator in the specific United States context. A deep history of slavery, post‐abolition segregation, welfare reform, and disproportionate imprisonment, alongside related structural inequities, contributes to African American children facing disproportionately higher rates of poverty than White children (Kravitz‐Wirtz et al., 2022; Miller, 2013; Roehrkasse, 2021). Consequently, any correlations between these demographic variables and STS should be interpreted strictly within that socio‐historical context.

Although the current analysis provides insufficient evidence to support the hypothesis that school‐level poverty alone predicts STS among school staff, we caution against dismissing school‐level poverty as a potential antecedent. Research posits that poverty and hardships related to limited material resources are tied to trauma and PTSD (Holmes et al., 2021). Additionally, significantly higher disciplinary referrals for subjective behaviours (such as being disrespectful) are made against Black students versus White students (Blitz et al., 2016), which may indicate an interaction among student poverty, trauma exposure, and staff's perception of their behaviour. In other words, poverty status and racial demographics of the school could themselves be correlates of a deeper issue with racial disparity in trauma exposure and perceived behaviour. A meta‐analysis by Hensel et al. (2015) adds depth to this, suggesting that the proportion of traumatized clients therapists have, or the amount of time spent working with them, may have a greater effect than simply the caseload size. Hence, it may not be a matter of classroom size or student population size, but rather the specific ratio of traumatized to non‐traumatized students that poses the most significant risk of STS to school staff. Recognizing these potential comorbidities means that schools serving higher poverty communities may require increased support for their staff.

Due to variation in effect direction and the diversity of constructs investigated, the Personal and Professional Qualities category does not yield a consistent pattern across all variables. This is largely a result of conceptual fragmentation: one study (1) investigated five work stressors and professional variables simultaneously, while the other nine examined only one or two. Furthermore, constructs were often inconsistently defined across the studies. For instance, staff ‘commitment’ and ‘investment’ were investigated in separate studies with differing conceptualizations. A similar disparity appeared in Study 19, which operationalized ‘engagement’ across three sub‐scales, whereas Study 43 focused on a profession‐level perception of commitment.

Where variables were more consistently defined in this category, some patterns emerged. Difficulties with time management, discipline and motivation, and general work‐related hazards were moderately positively associated with STS across the three studies that examined them. While these variables may warrant attention as potential targets for intervention, the limited number of studies and lingering inconsistencies in conceptualization caution against treating them as established explanatory factors. Future research should prioritize consistent operationalization and, where appropriate, account for construct overlap to clarify these professional relationships.

### STS instruments

The almost‐universal use of the ProQOL (Stamm, 2010) and, to a lesser extent, the STSS (Bride et al., 2004), reflects an STS measurement landscape inherited from clinical nursing and social work rather than developed for educational contexts. While three studies utilized school‐specific tools, these remain largely grounded in interpretations of original clinical measures like the STSS. As argued by the author of the TSTSS, such models often excluded critical ‘negative changes’ associated with STS (Schepers, 2017, p. 50), which formed the rationale for developing their school‐related measure. This potential over‐dependency matters, as these dominant measures in the field carry well‐documented limitations in educational settings, such as weak construct validity, conceptual overlap with burnout (particularly for the ProQOL), and a lack of specific norming for educational populations (Heritage et al., 2018; Ormiston et al., 2022). Such limitations suggest that current instruments may underestimate educator‐specific trauma or overestimate STS prevalence by conflating it with general occupational burnout. While the reviewed studies provided instrument validity justifications, these findings underscore the need for future research to investigate how educators qualitatively experience secondary trauma to ensure that structural factors of future measures are ecologically valid and consistently used.

### Limitations

Several limitations were considered. First, although our QuADS appraisal indicated that the studies were of acceptable to good quality, the inclusion of PhD dissertations/theses, which are critical for enhancing the depth of the inquiry, may introduce greater variance in methodological quality. Therefore, a measured approach should be taken when interpreting these results. Second, due to the state of the literature, comprising heterogeneous variables and analysis methods across studies, we were only able to indicate patterns and enumerate statistical significance and effect sizes. This approach is limited. Accordingly, future research may want to utilize sections of this review to conduct meta‐analyses, focusing on specific risk or resilience factors. Third, the lack of longitudinal analyses and insufficient exploration of interactions among all explanatory factors should be considered. Our findings present associations and maps patterns of evidence, and conclusions about generalizability and causality should be avoided. Fourth, approximately 77% of the studies were conducted in the United States, creating a significant imbalance in population‐related extraneous variables. For instance, the unique nature of trauma from widespread school shootings could influence direct and secondary trauma exposure in US populations (Jowarder, 2023) and was not accounted for in this analysis. Finally, many recent studies were conducted during the COVID‐19 pandemic, a period of unprecedented stress for educators, which may have acted as a significant confounding variable that was not explicitly measured in most included studies.

### Implications for practice and future research

To the best of our knowledge, this is the first attempt to systematically chart and synthesize the explanatory factors of STS specifically in school personnel, mapping evidence across six theoretically grounded predictor domains and tabulating the instruments used to assess STS in this population. The systematic tabulation of measurement instruments reveals a field‐wide reliance on tools not designed for educational populations; a finding with direct implications for how future research in this area might be designed, evaluated, and interpreted. Altogether, these findings provide an evidence base that can inform both school‐level policy and future research priorities in the field.

The practice implications of these findings are noteworthy. The evidence identified in this review suggests that fostering staff's adaptive cognitive and emotional resources, such as emotional intelligence, regulation, and cognitive reappraisal, may help to promote resilience to STS. While activities that bolster job meaning can be self‐initiated or implemented through professional development, a significant contribution of this work is the emphasis on systemic change. Interventions must move beyond a sole focus on individual self‐care to adopt organizational approaches that cultivate trauma‐informed cultures around staff's' well‐being. In such environments, leadership plays a critical role by actively promoting support‐seeking and providing structured professional support. Where feasible, schools could potentially improve personnel well‐being and student outcomes by prioritizing these steps, possibly within a tiered structure that targets the most highly exposed staff.

This review reveals several potential paths for advancing the field. Future research should prioritize longitudinal designs to explore temporal relationships and interactions between variables such as grade level, job role, and school‐level poverty. There is also a distinct need for more research conducted beyond the United States to address current geographical imbalances and for studies employing intersectional analyses to understand the experiences of staff from diverse racial, gender, and socioeconomic backgrounds. Furthermore, attempts to conceptualize variables homogeneously could resolve existing inconsistencies regarding SES and professional qualities. Finally, as a comprehensive understanding of the efficacy of trauma‐informed policies in schools remains incomplete (Maynard et al., 2019), developing a valid measure specifically for the ecological context of schools is essential. Establishing such a standardized metric would significantly advance the field by enabling researchers to assess more accurately the impact of trauma‐informed implementation on reducing STS among school staff.

## CONCLUSION

This scoping review mapped and synthesized the evidence on risk and resilience factors for STS in school personnel. Several factors emerged as potentially important associations that warrant further investigation. However, the most strongly supported individual‐level explanatory risk factors were frequent exposure to student trauma and a history of personal trauma, each showing moderate‐to‐large effect sizes across the majority of investigating studies. In contrast, robust protective effects emerged for cognitive and emotional personal resources, such as resilience, adaptive coping, problem‐solving orientations, and emotional intelligence. At the school organization level, the most robust protective factor surrounds proactive leadership support embedded within trauma‐informed organizational cultures that promote support‐seeking. These findings could potentially influence policy, interventions, and future research in the school context.

## AUTHOR CONTRIBUTIONS

**Paul Gordon:** Conceptualization; investigation; formal analysis; writing – original draft; methodology. **Lisa Jane Rodgers:** Formal analysis. **Martin Dempster:** Supervision. **Donncha Hanna:** Supervision; writing – review and editing; conceptualization; methodology.

## CONFLICT OF INTEREST STATEMENT

The authors declare no conflicts of interest.

## Supporting information


Appendix S1.

## Data Availability

The data that support the findings of this study are available in the tables and supplementary material of this article.
